# Atlanta Medical and Surgical Union

**Published:** 1885-03

**Authors:** 


### ATLANTA MEDICAL AND SURGICAL UNION.

Reported for THE ATLANTA MEDICAL AND SURGICAL JOURNAL.

STATED MEETING, January 20th.

Dr. J. D. Wilson, President, in the Chair.

Dr. J. McF. Gaston, by request, reported

A CASE OF STRANGULATED HERNIA, as follows:

Having recently attended, in connection with two of your members, upon a most interesting case in which there were many obscure complications, I have to report it in compliance with the request of my colleagues in the treatment of the case. Having some points of much practical importance, I would state at the outset, that the patient was in the immediate charge of Dr. W. B. Parks, who informed me that he was called on Friday, December 26, 1884, to see this lady, about fifty-two years old, suffering from troubles in the pelvic region, which she referred to the womb. She had her bowels moved by Epsom salts previously. He gave her a hypodermic injection of morphine which afforded relief, and she was not seen on the following day, Saturday; but being summoned on Sunday, he found the patient with great pain in the bowels and also gastric pains, accompanied by vomiting. His attention was called also to an inflamed lymphatic gland in the right groin for which a poultice was ordered; and an enema was likewise directed; but on returning in the afternoon, he found that no effect had followed, and he enjoined its repetition. Upon visiting the patient the next day, Monday, the 29th, there had not still been any evacuation from the bowels, and the vomiting continued with some fever, the temperature being 100 degrees. He gave two enemata at the interval of the morning and afternoon visits, and prescribed infusion of senna internally in small doses frequently repeated. But no evacuation from the bowels occurred during the day or night, excepting a few round, hard masses which returned with the enemata, thus completing four days without a motion. On Tuesday the inflamed lymphatic gland in the groin was still painful and the vomiting continued, when I was called in consultation by Dr. Parks. Upon seeing the patient in the afternoon of the 30th of December, 1884, I was informed by Dr. Parks that the symptoms indicated intestinal obstruction, and very soon after entering the bed-room, the patient had an access of vomiting, when a considerable quantity of gross fluid, with a dark greenish hue, was brought up; and afterwards she expressed herself as being very much relieved, resting upon her back, which she had not been able to do previously without discomfort. Upon making a general examination there was little indication of constitutional excitement, the pulse being soft and but little more frequency than natural, while the temperature was normal. But there was considerable typanitic distention of the abdomen and some tenderness, especially in the right iliac fossa; while there was an enlarged and indurated lymphatic gland located in the inguinal region, and corresponding to the outer opening of the inguinal canal, as near as could be determined by the manipulation that was admissible in the sensitive condition of the parts.

A repetition of the enema still failed to bring away anything but a few scybile. With a view to allay the irritability of the stomach the following was prescribed by us for the night:

℞. Lime water, 3 ounces, camphor water 2 ounces and peppermint water 1 ounce. Mix and take a tablespoonful every half hour while vomiting or nausea continues.

As a local application over the inflamed inguinal gland belladonna ointment was ordered, and to be used also as an embrocation over the lower bowels.

December 31st. The patient has passed a comparatively comfortable night, and taken some tea with milk without vomiting, and yet there was no opening through the intestinal canal; so that she was ordered an enema consisting of two ounces of castor oil and a teaspoonful of common salt, with a half pint of corn meal gruel, and if the first failed to secure an evacuation, to be repeated within two hours.

As there was some increase in the swelling of the lymphatic gland with more sensibility upon any attempt at manipulation, a poultice was directed over the coating of belladonna ointment in the inguinal region.

There was less distention and tenderness of the abdomen than on the day previous and no febrile excitement, so that the lime-water mixture was continued in doses of a teaspoonful every two hours as a simple placebo.

January 1st, 1885. The patient was seen at nine o'clock in the morning, in company with Dr. Parks, and finding that no passage, excepting some scybala, had resulted from the castor oil and salt gruel enema of the previous afternoon, she was ordered the following:

℞. Saccharated calomel, nitrate of potash, *aa* 20 grains. Mix and divide in two papers. Take them with an interval of two hours.

The increase of the inflammation in the inguinal region called for a resolvent, and we prescribed:

℞. Mercurial ointment 1 ounce, camphor 1 drachm. Mix and foment over the swelling, using the belladonna ointment as an embrocation to the bowels.

Dr. Parks reported in the evening that a repetition of the enema some hours after taking the calomel and nitre had induced a proper fœcal evacuation from the bowels, which was free from the mucus that had accompanied the scybala on the previous occasions.

January 2d. In the absence of Dr. Parks from the city I saw the patient in the morning, and finding some febrile excitement with increase of local inflammation, so that the tissues of the thigh below the inguinal line or crease were raised and reddened, and that the abdominal distention and tenderness had increased, it seemed best to have assistance before determining upon further measures of treatment.

Dr. J. A. Gray was invited to see the case with me in the afternoon, and as the fever then indicated aggravation, with a pulse of 128 to the minute, and temperature 102 degrees, with considerable swelling and sensitiveness in the inguinal region, while the abdominal tenderness and distention still continued, we ordered for the relief of the total suspension of the intestinal action the following laxative:

℞. Compound infusion of senna, 6 ounces. Take a wineglass full every two hours until it operates, or all is taken to-night.

And she was directed to continue the local application of the belladonna and camphorated mercurial ointment. My explanation of the symptoms up to this time was that a strangulated hernia had existed previous to my first visit, on which occasion it was partially released by the act of vomiting, but that adhesions had taken place around the internal ring, from which point inflammation extended along the canal producing the enlargement of the lymphatic gland corresponding to the external ring above Poupart's ligament, which was noted upon my first examination of the case. It was supposed that the lumen of the intestine was sufficiently open to admit of the passage of its contents, as observed in the evacuation after taking the calomel and using an enema on the day previous, and that the transmitted irritation would be lessened by a free discharge from the bowels.

January 3d. Upon visiting the patient alone in the morning, I found that the bowels had been well moved during the night with apparent relief to the symptoms, the pulse being now only 110 to the minute and temperature 100 degrees. But the tongue presented a dry, brown streak along its central line and there was some abdominal tenderness, though the typanitis had disappeared and I prescribed the "Perules de essence d'terebenthine du clertan" of which two capsules should be taken every three hours, with a continuation of the local applications.

At my afternoon visit found no material change in her condition, excepting some discomfort of the bowels, for which she was directed to take a teaspoonful of paregoric at night.

January 4th. Dr. Parks having returned, we saw the patient together at 9 o'clock in the morning, finding the pulse 120 to the minute and temperature 102 degrees, with deep red discoloration extending from the right inguinal across the iliac to the lumbar region. We prescribed internally:

℞. Acetate of ammonia, 6 ounces; to be taken in teaspoonful doses every hour, and to be used externally, as follows:

℞. Acetate of lead, ʒi, acetic acid f ℥i, acetated tincture of opium f ʒiv, water f ℥x. Mix and apply upon flaxseed poultices over the entire surface within the crest of ilium.

She had taken some light nourishment of a fluid nature, but no solid food at any time since the vomiting ceased on the 30th ult; and she was directed to use the wine whey, made by adding four ounces of Madeira wine to eight ounces of boiling milk, of which a wineglass was to be taken every hour, after the dose of acetate ammonia.

January 5th. We encountered an aggravation of all the symptoms, with loss of vital force, accompanied by an erysipelatous appearance extending on the right side from the inguinal to the lumbar region, which was previously inflamed. In view of the failing powers of the organization, the following prescription was made for internal use continuously:

℞. Sulphate of quinine, 1 drachm; muriated tincture of iron, 1 ounce; water, three ounces. Mix and give a teaspoonful every two hours with a little water. And for correction locally and generally we applied injections, hypodermically, of the following:

℞. Carbolic acid, 1 drachm; glycerine, 1 ounce; water, 2 ounces. Mix and introduce in various parts, including the labia majora on the right side. The flaxseed poultice was continued to the entire surface of inflamed parts. She was directed to take milk, soft eggs, beef tea or other nutritious food which might be acceptable to her stomach.

The wine was recommended pure, or brandy in the form of milk punch.

January 6th. The pulse was 130 to the minute and temperature 102, with disintegration of the tissues at two points nearly corresponding to Poupart's ligament, and marked general prostration, but the intellectual faculties were unimpaired. As she complained of much thirst a tablespoonful of acetate of ammonia was given in the hour that intervened in the regular periods for the tincture of iron and quinine. Subcutaneous injections of the carbolized solution were made within the crest of the ilium, and it was thrown freely into the two openings existing in the inguinal region.

January 6th. Dr. J. A. Gray saw the case with Dr. Parks and myself in the afternoon and concurred in the continued use of the tincture of iron and quinine with the poultices, after having repeated the injections of the carbolized solution not only hypodermically but in two open orifices from disintegration of the tissues in the inguinal region; and recommended porter, which was given.

There had been throughout a local inflammation of this part, which had passed into a gangrenous state of the surrounding tissues.

January 7th. Being summoned to meet Dr. Parks at the house of our patient, whither he had been called prior to the hour marked for our meeting there, she was found speechless and pulseless, for the relief of which my colleague had already resorted to subcutaneous injections of brandy, and I had only to suggest the trial of sulphuric ether by the same process, but without the least expectation on the part of either of us that she could survive until the close of the day.

She expired at 4 p. m.; and about the same hour on the following day, January 8th, Drs. Gray and Parks coöperated with me in making the autopsy.

The diagnosis submitted to my colleagues prior to making the post-mortem examination was verified, except in one point, which it was impossible to distinguish in advance, under the circumstances of the case. Upon laying open the lower abdomen by three linear incisions from the umbilicus, one being to the os pubis and the other to the spinous process of the ilium on either side, there was but little evidence of peritoneal inflammation, and yet in the internal femoral ring on the right side a knuckle of the ilium had been secured by adhesions and the contained portions of the intestinal wall had given way so as to admit of the passage of the contents of the intestines into the femoral canal, which penetrated into the deeper seated tissues and propagated the inflammatory action to the inguinal glands, ultimately producing disintegration of the cellular tissue and gangrene in the adjacent parts. The death was doubtless from septicæmia as a result of the septic contamination set up by the free exudations of the contents of the small intestines at a point about fifteen feet in the course measured from the ilio-cœcal connection.

The attachment of the outer walls of the ileum to the inner surface of the femoral ring was supposed to have yielded so much in the act of vomiting, upon my first visit to the case, as to relieve the patient of the symptoms of occlusion in the intestinal canal, and to have admitted of a sufficient lumen for the passage of its contents; and yet with an aperture on one side, admitted of the entrance of such matter into the femoral canal and thence by a disintegrating process into the cellular tissues around.

By a retrograde process the portion of the wall of the ileum which adhered around this internal femoral ring, had become involved in the gangrenous degeneration to such an extent that in lifting up this loop of the intestine to exhibit the adhesions to my colleagues, it yielded at one point, and there was an escape of a small portion of the dark contents of the bowel into the peritoneal cavity, which, however, had not occurred previously; and thus the peritoneal membrane was very slightly, if at all, inflamed. The death was not, therefore, attributable to peritonitis, but to blood poisoning and degeneration of the tissues from the penetration of the fœcal matter into the vital structure.

Dr. Noble asked Dr. Gaston why he did not operate sooner. Dr. Gaston replied that after his first visit, there was never a symptom of strangulation, the vomiting ceased, the bowels responded to cathartics, and there was nothing to lead him to suspect the true condition of the bowel.

Dr. Gray corroborated Dr. Gaston's statements.

Dr. G. H. Noble reported a case of

EARLY OPERATION WITHOUT OPENING THE SAC IN STRANGULATED INGUINAL HERNIA.

Dr. Noble stated that the hernia occurred in a well developed man, and had existed for one hour and a half before he saw him. He found the tumor about the size of a cocoanut.

The man had made every effort to reduce the hernia before sending for medical aid. He found the patient suffering with every symptom of strangulated hernia, except the vomiting.

With the assistance of Dr. Curtis, every effort was made at reduction, even to placing the patient completely under the influence of an anæsthetic. Failing in this an operation was decided upon. With the assistance of Drs. Hall, Wilson and Elkin, we made the usual operation, dividing the tissues down to the sac, when the external pilla of the ring was cut, then with considerable force the intestines were returned without opening the sac. The operation was made four and a half hours after strangulation occurred.

At no time since the operation has the patient's temperature been above 100¾ degrees Fahr., or his pulse above 78 per minute. This mode of operation places the patient in very little danger, since no blood or foreign substance gets into the abdominal cavity.

Dr. Gaston asked if it was a recent hernia.

Dr. Noble replied that it was not, that the hernia had existed for quite a while, but had suddenly become strangulated on the day of operation.

Dr. Noble asked if he was justifiable in resorting to operative procedure so soon after operation.

Dr. Gaston stated that in view of the fact that Dr. Noble had tried every means of reduction and failed, he thought the operation justifiable.

Dr. Elkin said, while the operation in this case might have been justifiable, that he thought in most instances it would be better to wait a longer time, and try every method of reduction before operating. He gave a case illustrating his views where the strangulation had existed for eight hours and which seemed to baffle the skill of every one at reduction, but which was finally reduced without operation.

Dr. Greer reported a case of

POST-PARTEM HEMORRHAGE, which was only controlled by the injection of hot and cold water into the cavity of the uterus, with hypodermic injections of ergot. The case developed into a puerperal fever for which he resorted to injections of solution of bi-chloride of mercury, one to two thousand in water. The irrigation of the cavity of the uterus was always attended by a marked reduction of the temperature. The patient was put on a supporting treatment and made a good recovery.

Dr. Gaston asked Dr. Greer if he had used a return tube for the water, when irrigating the wound.

Dr. Greer replied that he had not, as the womb was sufficiently dilated as not to require it.

Dr. W. B. Parks reported a case of partial occlusion of the vagina relieved by dilatation.

Dr. J. C. Avary reported a case of urinary calculi, and exhibited a number of calculi that had been passed from the urethra of the patient. Dr. Avary was requested to present a more detailed account of the case at the next meeting.

